# Investigating brain activity at rest in patients with persistent genital arousal disorder (PGAD) using functional magnetic resonance imaging

**DOI:** 10.1038/s41598-024-82695-y

**Published:** 2025-02-11

**Authors:** Eleni Dalkeranidis, Franziska M L M Kümpers, Christopher Sinke, Tillmann H C Krüger

**Affiliations:** 1 Division of Clinical Psychology and Sexual Medicine, Department of Psychiatry, Social Psychiatry and Psychotherapy, Hannover Medical School, Carl-Neuberg-Str. 1, 30625 Hanover, Germany; 2 Department of Psychiatry and Psychotherapy, Medical Faculty, LVR-Klinikum Düsseldorf, Heinrich-Heine-University, Duesseldorf, Germany; 3 Center for Systems Neuroscience, Bünteweg 2, 30559 Hanover, Germany

**Keywords:** Persistent genital arousal disorder (PGAD), Neuroimaging, Resting-state, Functional connectivity, Women, Magnetic resonance imaging, Urogenital diseases

## Abstract

Persistent genital arousal disorder (PGAD) is a rare disease causing high emotional distress eminently impacting the individual’s quality of life. Experts in this field assume that the disease is caused by a multifaceted interplay of different etiologies which may share a common neurobiological basis. However, only one functional neuroimaging investigation exist, and a more in-depth comprehension of the neurobiological foundation is required. Therefore, this study aims to provide new insights into how the functional integration of brain regions may relate to PGAD. By using the functional magnetic resonance imaging (fMRI) technique, functional connectivity at rest (rs-FC) was compared between patients suffering PGAD (n = 26) and healthy controls (n = 26). Patients with PGAD showed different pattern in connectivity within brain structures putatively associated with the psychological and somatic dimensions of the disease including the right amygdala, left anterior cingulate cortex, right insula cortex, thalamic nuclei and prefrontal regions as seeds. The majority of these showed differences in brain connectivity pattern to the precuneus and prefrontal regions. The study offers preliminary insights into the characteristics and relevant neural mechanisms of PGAD. Nevertheless, since this study did not identify any peripheral correlates that would corroborate the interpretation of these findings, they were interpreted from a more theoretical perspective, thereby offering potential areas of focus for future research.

## Introduction

Persistent genital arousal disorder (PGAD) is a disease characterized by unwanted and intense genital arousal. Arousal is often described as "being on the verge of an orgasm"^1^. However, the manifestation of the symptoms can also be highly heterogeneous (e.g., buzzing,pain, itching)^1,2,4^. The disorder is mainly described in cisgender (gender identity equals the sex assigned at birth) women^1^. While most patients experience symptoms in the clitoris, the location of the sensations can also manifest in other genito-pelvic regions (e.g., mons pubis, vestibule, and vagina)^2^. Therefore, some researchers propose adjusting the name to "persistent genital arousal disorder/genito-pelvic dysesthesia"^1^. To maintain consistency throughout this manuscript, the term PGAD will be used throughout.

The International Society for the Study of Women’s Sexual Health (ISSWSH) issued an expert consensus in 2021 containing generally accepted diagnostic criteria and formulated a therapy algorithm based on the current state of knowledge^1^. In this, *diagnostic criteria* are defined as: “(1) persistent or recurrent, unwanted or intrusive, distressing sensations of genital arousal (2) duration of ≥3 months (3) may include other types of genito-pelvic dysesthesia (e.g., buzzing, tingling, burning, twitching, itch, pain (4) most commonly experienced in the clitoris but also in other genito-pelvic regions (e.g., mons pubis, vulva, vestibule, vagina, urethra, perineal region, bladder, and/or rectum) (5) may include being on the verge of orgasm, experiencing uncontrollable orgasms, and/or having excessive number of orgasms (6) not associated with concomitant sexual interest, thought, or fantasies”^1^.

PGAD causes high emotional distress negatively impacting the affected individual’s quality of life^3,4^. Anxiety, depression, panic attacks and obsessive–compulsive disorder^2,3,5–7^ are common among patients with PGAD. According to Kümpers et al.^2^, psychiatric comorbidities tend to be mainly reported after the onset of the PGAD and are likely a result of heightened psychological distress caused by the disease.

Several theories about the potential etiologies and pathophysiology of PGAD exist. However, the ISSWH panelists assume the different etiologies may share a common neurobiological basis^1^. To date, there has only been a brief examination of neurological changes in individuals with PGAD as part of the ISSWSH consensus paper^1^. The researcher analyzed functional magnetic resonance imaging (fMRI) data of 3 women with PGAD and compared it with 12 healthy volunteers without PGAD. The healthy control group was instructed to think about clitoral stimulation without any applied physical stimulation while undergoing the scanning procedure. Altered brain activity within the thalamus (THA) and primary (SI) and secondary (SII) somatosensory cortex were observed in patients with PGAD. However, using the data as a reference is difficult due to the small group size and no detailed data publication.

Sexual behavior is a multifaceted process involving cognitive, motivational, emotional and autonomic/neuroendocrine components^8^. Even though there is a lack of sexual desire and significantly higher rates of sexual dysfunction^2^, the quality of arousal is often described by PGAD patients as being "on the verge of orgasm"^9^ with some patients even reporting the occurrence of uncontrollable spontaneous orgasms^6,10^. Therefore, it could be hypothesized that the neurological differences that underpin PGAD may occur within brain areas involved in the different dimensions of physiological sexual behavior for instance the prefrontal cortex (PFC), amygdala (AMY), insula or anterior cingulate cortex (ACC)^8^.

Research suggests that PGAD may be a phenotypic variation of Overactive Bladder Syndrome (OAB)^11,12^. Kümpers et al.^2^ examined 26 patients with PGAD, finding that 73.1% exhibited elevated rates of urinary urgency, while 46.2% demonstrated increased urinary frequency. These observations align with the symptoms commonly associated with overactive bladder syndrome (OAB)^2^. The authors hypothesized that there may be a possible overlap between bladder and genital function based on their common spinal representation (S2–S4)^2^.

Micturition is the act of urination^13^. Due to the urinary abnormalities observed in individuals with PGAD, one could reasonably infer that there may be alterations in the brain areas associated with micturition. For instance the PFC, which plays a pivotal role in the urinary control system, determining whether to initiate urination^14^. The thalamus (THA) serving as an integral subcortical relay station, transmitting incoming sensory stimuli, including those originating from the bladder^14,15^. The interoceptive and emotional component of micturition is mediated by the limbic system, including the ACC and bilateral AMY^14^. In addition, the insula is believed to be involved in bladder filling and voiding sensations^14^. Besides these four cortical subregions, supraspinal control of continence and micturition is believed to be centered within the pontine micturition center (PMC) and periaqueductal gray (PAG)^15–17^.

This neuroimaging study aims to gain a more in-depth understanding of the neurological basis of PGAD. As a starting point resting-state functional magnetic resonance imaging (rs-fMRI) in patients suffering from PGAD was analyzed and compared to healthy controls. Rs-fMRI facilitates revealing the brain’s intrinsic organization into distinct large-scale connectivity networks^18^. This method permits an investigation of multiple brain regions that may be implicated in diverse aspects of the disease’s presentation, as opposed to focusing on a single region of interest. As this is the first attempt to gain knowledge of the underlying neurological mechanism of PGAD, the study is exploratory in nature. The relatively unspecific hypotheses were developed by drawing insights from various sources including the results of the consensus paper^1^, investigations on brain-bladder control e.g.,^16,19^ and physiological sexual behavior e.g.,^20,21^. It was hypothesized that different pattern of brain activity would primarily occur within brain areas which have been linked to diverse symptoms of PGAD including changed sensorimotor control of the genitals, altered sexual perception or urinary abnormalities.

## Methods

The present rs-fMRI investigation is part of a larger project investigating PGAD called the ‘iPGAD study’, which was registered at ClinicalTrials.gov (Protocol ID: 8589_BO_S_2019; NCT04566783). The data collection period was from June 2020 to August 2021. The study was conducted according to the Declaration of Helsinki 1964, updated in October 2013 and the study protocol (Nr. 8589_BO_S_2019) was accepted by the Ethics Committee of the Hannover Medical School. A reimbursement of 50 Euros was given to each participant. Written informed consent was obtained from the participants before study participation. The study was funded by the *European Society for Sexual Medicine* (ESSM).

### Participants

**Fig. 1:**
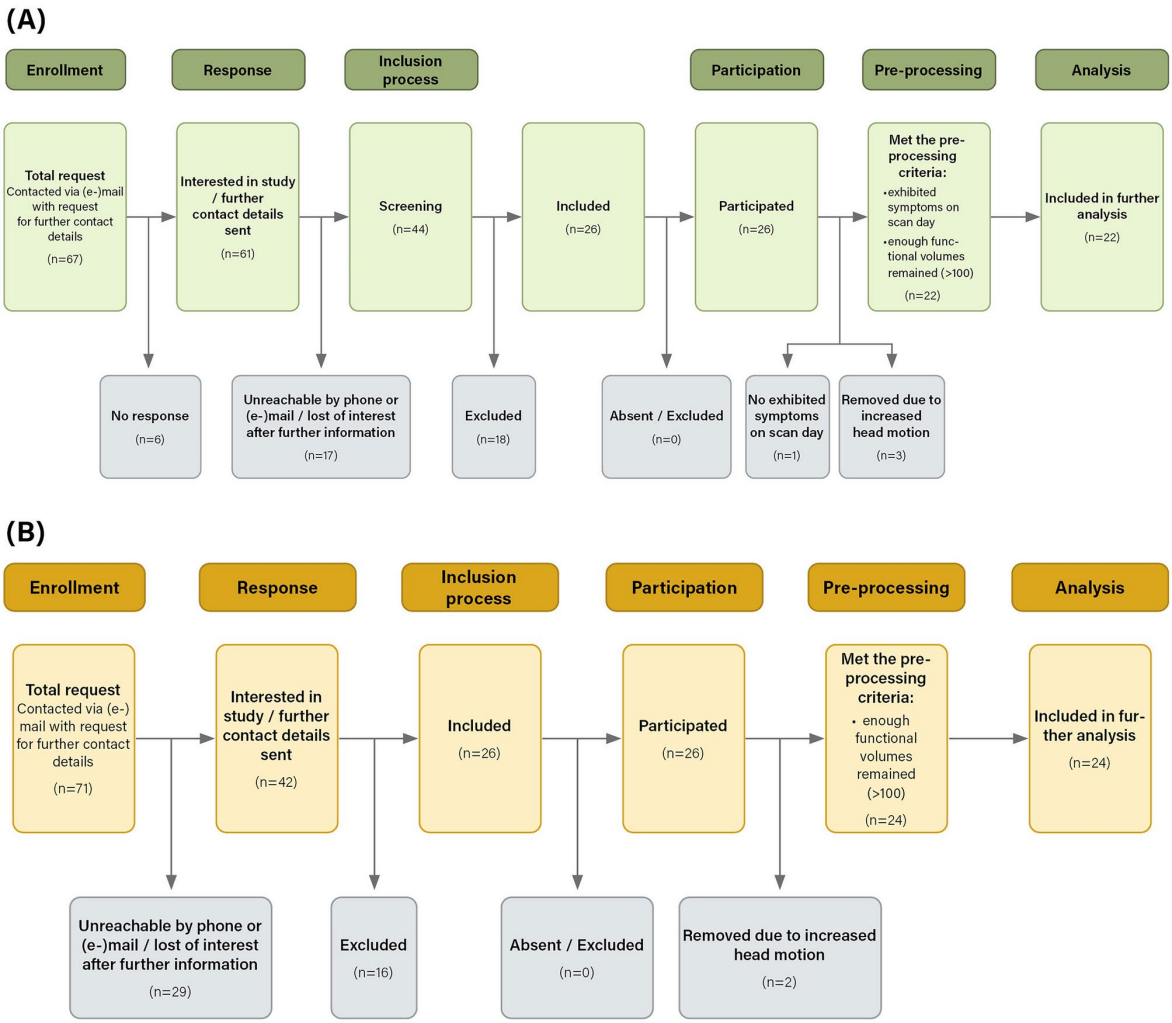
(**A**) Recruitment and pre-processing of PGAD group; (**B**) recruitment and pre-processing of healthy controls.

For the study 26 women suffering from PGAD were recruited who met the following diagnostic criteria stated by Leiblum and Nathan^22^ and as assessed by an experienced medical doctor and sexual medicine specialist (T.K.): (1) genital arousal symptoms do not subside entirely on their own and persist for an extended period (hours or days); (2) arousal symptoms do not resolve with orgasmic experiences or may require numerous orgasms to disappear; (3) genital arousal is unrelated to feelings of sexual excitement or desire; (4) sexual, nonsexual, or even no apparent stimulus may trigger symptoms; (5) symptoms are perceived as intrusive and uncontrolled. Healthy controls (HC; n = 26) were matched by age and academic degree. Most of the PGAD patients have been treated in the faculty’s sexual medicine ambulance (n = 19). The other PGAD patients were recruited via internet forums (n = 7). Recruitment of healthy controls was via the intranet of the Hannover Medical School (n = 14) and word of mouth (n = 12). Study exclusion criteria included severe and acute psychological and physical diseases and intellectual disabilities. For more details on the recruitment process, see Fig. 1. Of the participants, 22 PGAD patients and 25 HC were right-handed. As PGAD is a rare disease^23^, it was very challenging to find drug-naïve patients. To minimize the effects of central nervous system (CNS) acting medication on the brain activity, patients were requested to stop taking these drugs for one intake period, with the intention of conducting the brain scan when the trough level of the medication has been reached. All participants were screened for MRI contraindications before the scanning procedure and were free to withdraw from the study at any time. The study sample can be ascertained in more detail from the paper by Kümpers et al., which forms part of the ‘iPGAD’-study and focuses on the clinical and phenomenological characterization of PGAD^2^.

### fMRI data acquisition

A Siemens MAGNETOM Skyra 3 T system (Siemens MAGNETOM Skyra, Erlangen) with a 64-channel head coil was used for the scanning procedure. The scanning session started with acquiring the rs-fMRI images to minimize emotional or cognitive influences. A scan time of 10.2 min was chosen to ensure high-quality data and minimize noise. 470 volumes were obtained for each participant. Patients were instructed to keep their eyes open while looking at a fixation cross and maintaining a still position. The resting-state functional data were acquired using a gradient simultaneous multislice EPI T2* sensitive sequence (repetition time (TR) = 1.31 s, echo time (TE) = 36 ms, field of view (FOV) = 208 × 208 mm, flip angle = 64°, 78 slices per slab, 2 × 2 × 2 mm voxel size and an acceleration factor (AF) of 6. In addition, a T1-weighted MRI image as an anatomical reference was obtained (TR/TE = 5 s/2.98 ms, FOV = 240 × 256 mm, flip angle = 4°, 176 slices and 1 × 1 × 1 mm voxel size).

### Pre-processing of the fMRI data

SPM12 (Welcome Trust Centre for Neuroimaging, London, UK) and DPABI 2.3^24^ were used to process and analyze the functional volumes. Considering the instability of the initial signal and the participant’s adaption to the scanner, the first five initial images were removed. The remaining 465 rs-fMRI volumes were slice-time corrected, realigned, and co-registered to the reference T1-images for motion correction. After using unified segmentation^25^ on the anatomical images, the images were normalized to Montreal Neurological Institute (MNI) stereotactic space^26^ and resampled at a resolution of 2 × 2 × 2 mm^3^. The images were then bandpass filtered to eliminate scanner drift from the data (0.01–0.08 Hz). Nuisance covariate regression was performed, regressing out head movement using Friston24 and the global signal. Friston24-parameter motion correction refers to a regression with autoregressive models of motion, incorporating 6 head motion parameters, 6 head motion parameters one time before, and the 12 corresponding squared items^27,28^. This method was selected based on prior research indicating that higher-order models are more effective at removing head motion effects^29,30^. Head motion scrubbing was performed using frame-wise displacement (FD) with a threshold of 0.5 and deleted one time point before and two after the detected volume^31^. After the scrubbing procedure a comparable number of volumes was left in both groups (HC: 432 ± 67, PGAD: 403 ± 107, Mann–Whitney-U-test: U = 238.5, Z = − 0.567, p = 0.571). Before the statistical analysis, the images were smoothed with a Gaussian kernel of 4 × 4 × 4 mm^3^. During the pre-processing procedure, three PGAD patients and two HC were excluded from the analysis, as head motion scrubbing resulted in less than 100 volumes. One patient did not exhibit acute PGAD symptoms on the day of scanning. To maintain a homogenous sample, she was omitted from the analysis. Therefore, the final sample consisted of 22 PGAD patients and 24 HC. For more details on the pre-processing process, see Fig. 1.

### First- level analysis

Seed-based correlation analysis is a widespread approach to study resting-state functional connectivity. This hypothesis-driven method assesses functional connectivity by correlating resting-state time courses of a preselected region of interest (ROI), commonly known as the seed. These seeds can be defined using an anatomical basis based on predefined anatomical atlantes from open-access toolboxes (e.g., automated anatomical atlas (AAL)) or coordinates from previous literature. When using coordinates, a sphere with a diameter of 5 mm was used. The following ROI seeds were selected from the 3^rd^ edition of the automated anatomical atlas (AAL3)^32^ for use in this study. This includes thalamic nuclei such as the (1) the bilateral ventral posterolateral thalamic nuclei (Thal_VPL), which act as first-order thalamic relays, primarily involved in the transmission of sensory inputs from the periphery to the cortex^33,34^. In addition, higher-order thalamic nuclei such as the (2) bilateral intralaminar thalamic (Thal_IL) nuclei and (3) bilateral medial mediodorsal thalamic nuclei (Thal_MDm), which have been linked to the integration of sensory information across multiple cortical circuits influencing the individual’s arousal and consciousness levels^33^, have been defined. The (4) amygdala (AMY) due to its essential role in the emotional-cognitive modulation of incoming sensory input especially bladder stimuli^14^ and its capability to evaluate the emotional significance of incoming erotic stimuli and modulation of sexual drive^8^, (5) the anterior cingulate cortex (ACC) as a critical component of the evaluation of afferent sensory inputs^35^, its function within the micturition process^14^ and its involvement in the motivational aspect of sexual behavior^8,21^, (6) the insular cortex as a modulator of sexual drive, its capability to sense the tumescence of the erectile organs^8^ and as an integral part of micturition^16,36^. Moreover, (7) the gyrus rectus has been selected due to its involvement in inhibitory system of sexual behavior^21^. Besides the AAL3, the Brainstem Navigator^37^ was utilized to select (8) the periaqueductal grey (PAG) as a seed ROI for further analysis since it is an important part of the micturition process^16^.

Also, ROI seeds were selected using coordinates from previous literature such as the (9) right [− 18 − 38 63] and (10) left [19 − 37 57] clitoral representation of the primary sensory cortex (SI) from a study by Michels et al.^38^. The clitoral representation of the SI was selected as a ROI since it displayed alterations in brain activity in previous studies^1^ and most patients localize their unwanted genital sensations at the clitoris^1,12^. In addition, coordinates of (11) the right primary motor cortex (MI) [12 − 30 74] from the study by Georgiadis et al.^20^ were chosen to examine the clitoral motor control. From the same paper by Georgiadis et al.^20^, coordinates of other important regions that display alterations during clitorally induced orgasm in healthy women were also extracted, including (12) the left inferior temporal gyrus [− 52 − 28 − 30], (13) left middle/inferior frontal gyrus (lateral OFC) [− 38 44 − 16], (14) right superior temporal gyrus (anterior temporal pole) [32 8 −44], (15) deep cerebellar nuclei [− 10 − 48 − 22] and (16) left inferior temporal gyrus [− 42 − 12 − 38]. In order to define the (17) Pontine Micturition Center (PMC) as a ROI seed due to its role in the induction of micturition^17^, the coordinates [4 − 36 − 34] from a study by Tadic et al.^39^ have been utilized.

The BOLD signals were averaged across all voxels within the ROI. Next, the time series for each ROI seed of a participant was extracted. A seed-based connectivity map for each patient was derived by calculating the Pearson’s correlation coefficients between the defined ROI’s time series and the whole brain’s voxels. Before the group-level analysis, Fisher’s r-to-Z transformation was used to convert the correlation coefficients into Z-scores.

### Second-level analysis

To identify brain regions with significant rs-FC differences between groups, a random effect two-sample t-test was conducted to compare the individual Z-values voxel-wise. The threshold for the analyses was set to p ≤ 0.05 false discovery rate (FDR) corrected for multiple comparisons on the cluster level. In addition, patients (n = 8) taking CNS acting medications with high trough levels on scan day (e.g., duloxetine, citalopram or aripripazol) were included as a covariate in the second-level model estimation.

### Clinical assessment of symptom intensity and onset

Symptom intensity during the MRI scan accessed directly after the resting-state measurement and average symptom intensity in the last four weeks were assessed using a numeric pain rating scale (NRS) (on a scale of 0–10, with 0 = neutral and 10 = extremely intense level of symptoms). In addition, the disease duration was determined by asking for the year of symptom onset.

### Data analysis of non-imaging data

SPSS software Version 27 (IBM Corporation, Amonk, NY, USA) was used to analyze clinical and demographic variable differences. Parameter estimates of the functional connectivity between regions exhibiting differences between groups was correlated with clinical (i.e., intensity of symptoms, duration of disease, average symptom intensity in the last four weeks) in the group of PGAD patients. Correlations between functional and behavioral data were evaluated using Pearson’s correlation coefficient using a threshold of p < 0.05.

## Results

By using t-tests or χ^2^ tests, demographic features of the final sample of PGAD patients and HC showed no significant differences regarding age, handedness and education (p > 0.05). Results are summarized Table 1.

**Table 1:** Sociodemographic parameters.

Matching parameters	PGAD (n = 22)	HC (n = 24)	Statistics (p-value)
Age (years): M ± SD; [range]	37.18 ± 13.8; [19–69]	37.88 ± 13.6; [21–72]	t = 0.104 (p = 0.749)
Education (years): M ± SD	11.81 ± 1.5	12.12; ± 1.4	t = 0.687 (p = 0.412)
Handedness (left/right)	4/22	1/23	χ^2^ = 2.32 (p = 0.127)

*M* mean, *SD* standard derivation, *PGAD* patients with persistent genital arousal disorder, *HC* healthy controls.

### Rs-FC differences in brain region associated with sensorimotor control (seeds: Thal_MDM, Thal_IL, Thal_VPL, SI and MI (clitoral representation)

**Fig. 2:**
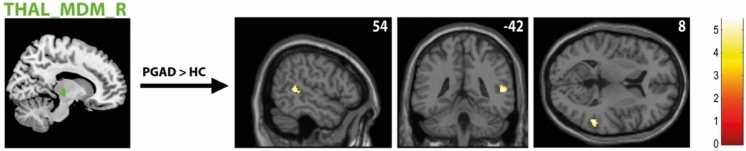
rs-FC of the right medial mediodorsal thalamic nucleus (Thal_MDM_R). Shown is the rs-FC of the right medial mediodorsal thalamic nucleus in the contrast (PGAD) > (HC) to the right middle/superior temporal cortex; (R) = right.

**Fig. 3:**
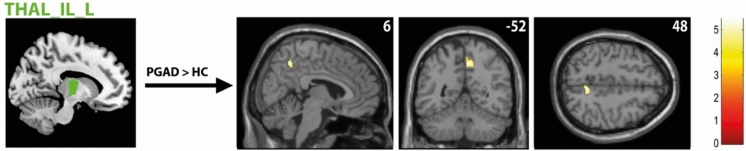
rs-FC of the left intralaminar thalamic nucleus (Thal_IL_L). Presented is the rs-FC of the left intralaminar thalamic nucleus in the contrast (PGAD) > (HC) to the right precuneus; (L) = left.

Group comparison revealed higher resting-state functional connectivity (rs-FC) between the right medial mediodorsal thalamic nucleus (Thal_MDM_R) and right middle/superior temporal cortex in the PGAD group (see Fig. 2). However, no significant intergroup differences between the left medial Thal_MDM and other brain structures were found. In addition, there was a heightened FC between the left intralaminar thalamic nucleus (Thal_IL_L) and the right precuneus in the PGAD group (see Fig. 3). No significant intergroup differences between the right Thal_IL and other brain structures were identified. There were no significant rs-FC differences between the bilateral ventral posterolateral thalamic nuclei (Thal_VPL), the clitoral representation of the SI, MI and other brain structures. See Table 2 for details.

**Table 2:** Group differences of resting-state functional connectivity using the contrast (PGAD) > (HC) and (HC) > (PGAD).

Seed ROI	Laterality	Connected cortical region	MNI			p-value*	k_E_	t-score	Parameter estimates (PGAD) M ± SD	Parameter estimates (HC) M ± SD
			x	y	z					
(PGAD) > (HC)										
Amygdala	R	Left ACC/left superior frontal gyrus, medial orbital	− 12	46	− 6	< .001	142	4.96	0.0833 ± 0.13	− 0.0569 ± 0.09
Insular cortex	R	Left precuneus	0	− 76	40	< .001	123	4.73	− 0.1145 ± 0.16	− 0.3137 ± 0.15
		Right cerebellum	36	− 56	− 42	< .001	217	5.19	− 0.0628 ± 0.11	− 0.2042 ± 0.10
Middle inferior frontal gyrus (lateral OFC)	L	Left precuneus	10	− 74	38	0.032	57	4.12	− 0.0444 ± 0.19	− 0.1770 ± 0.15
Superior temporal gyrus	R	Left precuneus	10	− 74	36	0.004	92	4.25	− 0.0037 ± 0.18	− 0.1407 ± 0.13
Inferior temporal gyrus	L	Left precuneus	0	− 78	42	0.026	71	4.92	− 0.0654 ± 0.21	− 0.2301 ± 0.15
Medial mediodorsal thalamic nucleus	R	Right middle/superior temporal cortex	54	− 42	8	< .001	71	4.87	0.1330 ± 0.10	0.0109 ± 0.12
Intralaminar thalamic nucleus	L	Right Precuneus	6	− 52	48	< .001	61	4.78	− 0.0001 ± 0.13	− 0.0923 ± 0.08
(HC) > (PGAD)										
ACC	L	Left middle frontal gyrus/left superior frontal gyrus, dorsolateral	− 26	40	18	0.045	56	3.99	0.1486 ± 0.16	0.3470 ± 0.15
PAG		Left Cerebellum	− 42	− 48	− 30	0.007	78	5.07	− 0.0615 ± 0.10	0.0743 ± 0.10

*ROI* region of interest, *MNI* Montreal Neurological Institute (x, left to right; y, anterior to posterior; z, dorsal to ventral), *k*_*E*_ cluster size, *PGAD* patients with persistent genital arousal disorder, *HC* healthy controls, *ACC* anterior cingulate cortex, *PAG* periaqueductal gray, *OFC* orbitofrontal cortex, *L* left, *R* right, *M* mean, *SD* standard derivation.

*The p-value is FDR-corrected on the cluster level.

### Rs-FC differences in brain regions associated with sexual perception and emotion regulation (seeds: AMY, ACC, gyrus rectus, left inferior temporal gyrus, left middle/inferior frontal gyrus (lateral OFC), right superior temporal gyrus (anterior temporal pole), left deep cerebellar nuclei)

**Fig. 4:**
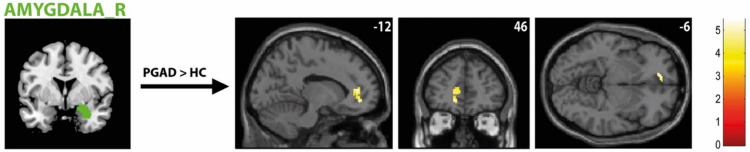
rs-FC of the right amygdala. Displayed is the rs-FC of the right amygdala in the contrast (PGAD) > (HC) to the left ACC extending into the left medial orbital part of the gyrus frontalis superior; (R) = right.

**Fig. 5:**
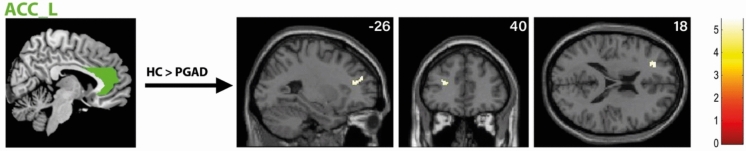
rs-FC of the left ACC. Shown is the rs-FC of the left ACC in the contrast (HC) > (PGAD) to the left middle frontal gyrus extending into the left superior, dorsolateral frontal gyrus; (L) = left.

**Fig. 6:**
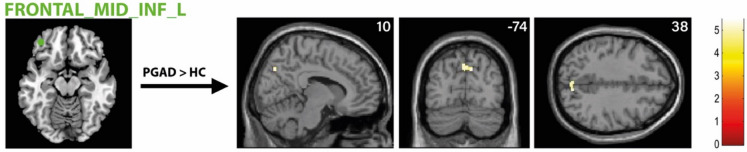
rs-FC of the left middle/inferior frontal gyrus/ lateral OFC (Frontal_Mid_Inf_L). Displayed is the rs-FC of the left middle/inferior frontal gyrus/lateral OFC in the contrast (PGAD) > (HC) to the left precuneus; (L) = left.

**Fig. 7:**
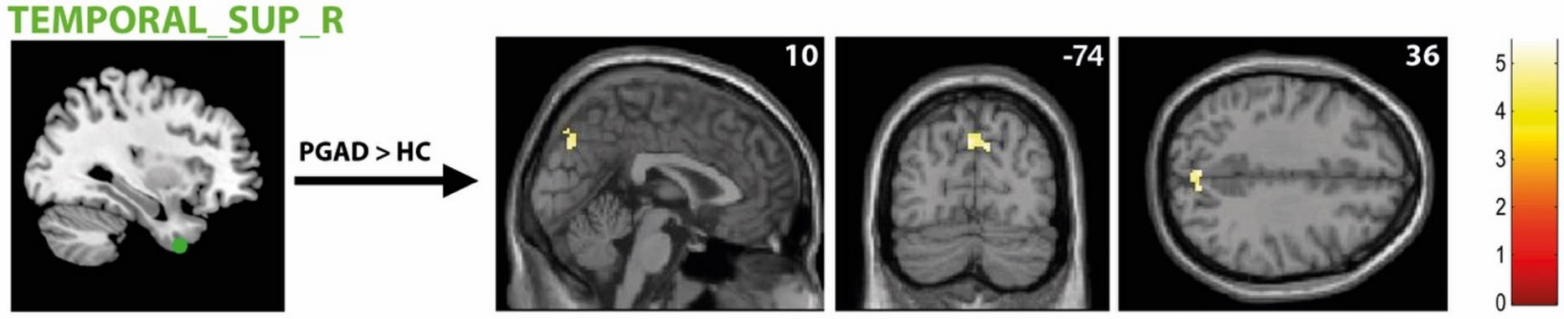
rs-FC of the right superior temporal gyrus (Temporal_Sup_R). Shown is the rs-FC of the right superior temporal gyrus in the contrast (PGAD) > (HC) to the left precuneus; (R) = right.

**Fig. 8:**
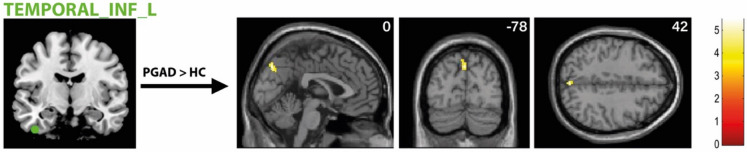
rs-FC of the left inferior temporal gyrus (Temporal_Inf_L). Shown is the rs-FC of the left inferior temporal gyrus in the contrast (PGAD) > (HC) to the left precuneus; (L) = left.

There was significantly higher rs-FC between the right AMY and left ACC extending into the left medial orbital part of the superior frontal gyrus in the PGAD group compared to HC (see Fig. 4). However, no significant intergroup differences between the left AMY and other brain structures were found. Conversely, there is diminished rs-FC between the left ACC and left middle frontal gyrus extending into the left dorsolateral superior frontal gyrus in PGAD patients (see Fig. 5). No significant rs-FC differences between the right ACC, bilateral gyrus rectus and other brain areas have been detected. Most of the brain regions associated with clitorally induced orgasm utilized from the paper by Georgiadis et al.^20^ showed higher rs-FC to the left precuneus, including the left middle/inferior frontal gyrus/lateral OFC (Frontal_Mid_Inf_L) (see Fig. 6), right superior temporal gyrus (Temporal_Sup_R) (see Fig. 7) and the left inferior temporal gyrus (Temporal_Inf_L) (see Fig. 8). However, no differences in pattern of brain activity had been identified between the left deep cerebellar nuclei and other brain regions. See Table 2 for details.

### Rs-FC differences in brain regions associated with micturition (seeds: insula, PAG, PMC)

**Fig. 9:**
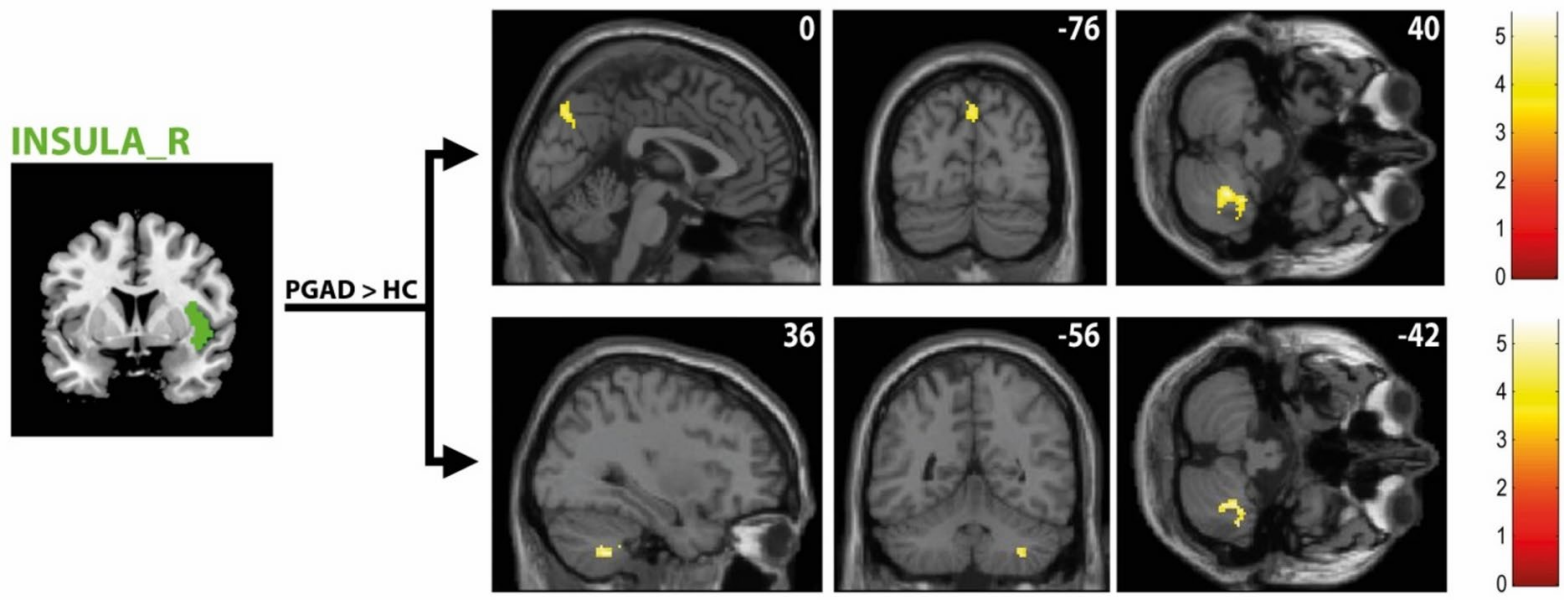
rs-FC of the right insular cortex. Shown is the rs-FC of the right insula in the contrast (PGAD) > (HC) to the left precuneus and the right cerebellum; (R) = right.

**Fig. 10:**
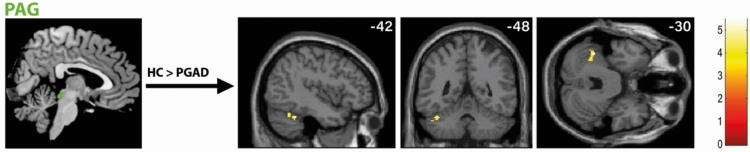
rs-FC of the PAG. Displayed is the rs-FC of the PAG in the contrast (HC) > (PGAD) to the right cerebellum.

Concerning the insula, rs-FC analysis on the right insular cortex reveled elevated connectivity to the right cerebellum in PGAD patients (see Fig. 9). In addition, there was a higher rs-FC between the right insular cortex and the left precuneus in PGAD patients. No significant rs-FC differences have been identified between the left insular cortex and other brain areas. Diminished rs-FC have been identified between the PAG and left cerebellum (see Fig. 10). No rs-FC differences have been revealed between the PMC and other brain areas. See Table 2 for details.

### Correlations between resting-state activity and clinical measures

Exploratory correlation analysis of the PGAD group was conducted to determine the relationship between the intrinsic connectivity of regions exhibiting significant group difference and clinical measures. The ROIs displaying significant group differences showed no correlation with disease duration and NRS scores of symptom severity during the scan and on average over the last four weeks.

## Discussion

The primary aim of this study was to determine differences in rs-FC in patients with PGAD. Rs-FC differences were found in brain areas potentially involved in the psychological and somatic dimensions of the disorder. However, the lack of correlation between the clinical and behavioral measures and brain regions exhibiting significant group differences in resting-state activity is unfortunate, but nevertheless a fact that must be acknowledged. It remains unclear whether the observed symptoms are a direct consequence of the different pattern of brain activity or whether they represent brain adaptations to other, as yet unidentified etiologies. Accordingly, this exploratory study aims to offer a theoretical interpretation of the results, thereby identifying potential avenues for future research. Furthermore, additional task-based studies and studies utilizing larger patient populations are required.

There are no significant group differences in FC within ROI seeds that were primarily associated with sensorimotor control such as the SI, MI or first-order thalamic relays like the VPL nuclei. However, besides the first-order thalamic relays, which mainly transmit sensory puts from the periphery to the cortex, there are higher-order thalamic relays, such as the medial and interlaminar nuclei that are reciprocally connected to the association cortex^33,40,41^ and receive minimal sensory input^33,40^. In higher-order relays the inputs mainly originate from various cortical and subcortical sources with excitatory or modulatory functions^40^. Based on this connectivity pattern, the intralaminar and medial thalamic nuclei are believed to be involved in various cognitive functions, such as attention, reward-based behavior, orientation and memory processing^33^. Saalmann^33^ suggests that intralaminar and medial thalamic neurons play a significant role in adjusting synchrony among different groups of cortical neurons based on behavioral demands. The author proposes a synchronization mechanism mediated by the thalamus that could integrate information across multiple cortical circuits influencing the individual’s arousal and consciousness levels^33^.

In the PGAD sample, the left intralaminar and right medial mediodorsal nuclei exhibited higher rs-FC with brain regions, which are involved in attentional processes (e.g., precuneus^42^) and in physiological sexual behavior (e.g., superior temporal cortex^21^). Hence, differences in rs-FC within the intralaminar nucleus could indicate an imbalance in the thalamic-mediated synchronization mechanism as discussed by Saalmann^33^. This alteration may be due to the need for extensive integration of information across diverse cortical circuits involved in an individual’s salience and sexual behavior. However, the underpinning reason for this imbalance remains unclear. Therefore, further investigations are required to validate this observation and explore peripheral correlates to determine the potential causes of the increased thalamic-mediated information processing.

As hypothesized, differences in rs-FC are observable in brain regions associated with different facets of sexual behavior. For instance, heightened rs-FC is observed between the left lateral orbitofrontal cortex (OFC) and the left precuneus. The precuneus is involved in modulating and integrating attentional processes^42–44^. Interestingly, the inhibitory mechanisms of sexual behavior are believed to be located in the prefrontal cortex (PFC)^8^. Georgiadis et al.^20^ observed heightened left lateral OFC activity in women instructed to refrain from experiencing orgasm during stimulation. The researchers suggested that intentionally regulating fundamental instincts, like sexual behavior, may be linked to the left lateral OFC’s activation^20^. Therefore, an elevated regional cerebral blood flow (rCBF) during stimulation could signify heightened inhibition, while a decreased rCBF during orgasm may indicate sexual disinhibition^20^.

Taking into account the left lateral OFC activity as a suppressor of sexual behavior and the role of the precuneus in attention modulation, the increased rs-FC between these regions may mirror an increased cognitive inhibitory mechanism. This potentially increased cognitive inhibitory mechanism may represent an adaptative response to the altered sexual perception in PGAD patients, aiming to suppress the persistent genital arousal and/or spontaneous orgasms. The theoretical foundation of this is based on frequent reports from individuals with PGAD who perceive the persistent genital sensations as shameful or embarrassing^5,45^ and are scared to experience an uncontrollable orgasm while being in public^46^. These reports underscore the need for patients to suppress these sensations effectively in order to uphold their daily functionality.

Carvalho et al.^47^ propose that personality traits, such as sexual conservative beliefs and lower openness, may mediate or sustain PGAD symptoms. Further research is encouraged to investigate this theoretical concept in greater detail. In particular, it would be beneficial to ascertain whether conscious suppression of symptoms, which may be influenced by personality traits, is associated with higher connectivity between the left lateral orbitofrontal cortex (OFC) and the left precuneus.

Another explanation for the higher connectivity between the left lateral orbitofrontal cortex (OFC) and the left precuneus may be a higher activation of the threat of negative consequences (SIS2) inhibition system proposed by Bancroft and Janssen^48^. Bancroft and Janssen postulate that there are two discrete inhibition systems responsible for the inhibition of sexual arousal. The first of these is the performance-related inhibition system (SIS1), while the second is the inhibition system related to the threat of negative consequences (SIS2)^48^. Kümpers et al. found that 19.2% of the 26 PGAD patients studied, reported masturbation and 26.9% identified sexual intercourse as triggering factors for their persistent genital sensations^2^. Therefore, it seems reasonable to hypothesize that the SIS2 may be more active to limit heightened sexual arousal, thereby reducing masturbation or sexual intercourse and preventing potential negative consequences, such as symptom worsening. In order to examine this hypothesis in more detail future research could employ the Sexual Inhibition and Sexual Excitation Scales (SIS/SES)^49^.

It is important to note that this study does not find any peripheral correlates to support these two sexual inhibition hypotheses. Therefore, a number of alternative interpretations for the different pattern of brain activity between the left lateral orbitofrontal cortex and the left precuneus are possible. It is thus recommended that the aforementioned hypotheses are taken as a potential starting point for further research.

Patients with PGAD exhibit significant heightened rs-FC in various regions of the brain implicated in the motor, cognitive, and emotional control of micturition. Interestingly, there is an elevated connectivity observed between the right insula, right cerebellum and the left precuneus. In individuals with normal bladder function, heightened responsiveness of the insula is observed as the bladder undergoes filling^36^ and is involved in integrating limbic and autonomic responses^50–52^. Both, the cerebellum and the precuneus, have been described to be involved in the initiation of voiding in healthy subjects^53^.

In addition, there is a stronger rs-FC between the right amygdala and left ACC extending into the left PFC. The ACC is involved in sensations of bladder fullness, micturition control, and pelvic floor contractions^36,54,55^, extending its role beyond sensorimotor functions to emotional and motivational aspects of micturition^52^. The activation of the PFC holds particular significance in determining if it is appropriate to initiate micturition^52^. However, rather than being a dictatorial, isolated decision center, the PFC seems to function as part of a network that not only processes sensory evidence for decision making, but also translates this evidence into an actionable response^52^. In summary, brain regions primarily involved in the interoceptive monitoring, emotional control and significance detection of the micturition process display higher connectivity.

PGAD patients have reported increased frequencies of urinary urgency^2,56^. Based on these reports and the different pattern of brain activity within brain parts involved in micturition, one can hypothesize that heightened sensory signals from the genitals may be misinterpreted as originating from the bladder due to a yet-unidentified mechanism. The anatomical basis for this theory would be the proximity of the bladder and genital areas in the spinal cord representation^57^, opening the possibility of some kind of cross-talk. The nerve pathways for these regions are closely intertwined, which could result in the overlapping sensation of needing to urinate and feeling sexually aroused. In this hypothesis, the stronger rs-FC between brain regions primarily associated with the significance and emotional control of the urination process can be understood as an elevated cognitive and emotional analysis and modification of the persistent sensation resembling urinary urgency.

It is important to note that this study did not investigate peripheral correlates. To assess the urgency symptoms of PGAD patients in more detail, questionnaires like the Overactive Bladder-Bladder Assessment Tool (OAB-BAT)^58^ could be employed. This approach would provide additional information that may support the interpretation offered. As the study population is not large enough to make statistically significant comparisons between patients with and without bladder symptoms, future studies with larger patient populations are encouraged to conduct this investigation.

In general, it is noteworthy that the precuneus, which plays a role in attentional processes^42^, appears to exhibit different pattern in connectivity with numerous selected ROIs of this study. This observation may indicate that the persistent genital sensations be attentionally processed and integrated within various brain regions. In other words, the persistent genitals sensations may repeatedly emerge from the subconscious into the conscious state due to yet unidentified mechanisms. It would be of interest to ascertain whether future research can validate these findings, which could help to gain a more holistic understanding of the brain connectivity pattern in PGAD patients.

## Limitations

The current study has some limitations. Firstly, the patient sample displayed highly heterogeneous clinical presentations of PGAD. Secondly, PGAD is a rare disease; therefore, it was challenging to find medicine-naïve participants. Although an attempt was made to conduct the study when medication trough level was reached and medication with high trough levels after one intake period were included in the second-level analysis, it is still possible that the CNS acting medication has an effect on the outcomes.

## Conclusions

The present study provides preliminary insights into the characteristics and possible neural mechanisms of PGAD. Rs-FC differences were found in brain areas potentially involved in psychological and somatic aspects of this disorder. The study was unable to ascertain the relationship between the intrinsic connectivity of regions exhibiting significant group differences and clinical correlates. Therefore, the results were interpreted in a more theory-oriented way thereby providing potential starting points for future research.

## Data Availability

The datasets generated during and/or analyzed during the current study are available from the corresponding author on reasonable request.
